# Supplementary material for: Biomimetic Inorganic Nanovectors as Tumor-Targeting Theranostic Platform against Triple-Negative Breast Cancer
Source: Pharmaceutics. 2023 Oct 22;15(10):2507. doi: 10.3390/pharmaceutics15102507 (PMC10610067; doi:10.3390/pharmaceutics15102507)
Supplement: Supplementary file 1 [file pharmaceutics-15-02507-s001.zip › pharmaceutics-2625153-supplementary.pdf]

Biomimetic Inorganic Nanovectors as Tumor-Targeting Theranostic Platform against Triple-Negative Breast Cancer

Huang Wen [1], Pekka Poutiainen [2], Enkhzaya Batnasan [3], Leena Latonen [3], Vesa-Pekka Lehto [1,*] and Wujun Xu [1,*]

1 Department of Technical Physics, University of Eastern Finland, Yliopistonranta 1F, 70211 Kuopio, Finland; hwen@uef.fi
2 Kuopio University Hospital, University of Eastern Finland, Puijonlaaksontie 2, 70210 Kuopio, Finland; pekka.poutiainen@pshyvinvointialue.fi
3 School of Medicine, University of Eastern Finland, Yliopistonranta 1F, 70211 Kuopio, Finland; enkhzaya.batnasan@uef.fi (E.B.); leena.latonen@uef.fi (L.L.)
* Correspondence: vesa-pekka.lehto@uef.fi (V.-P.L.); wujun.xu@uef.fi (W.X.)

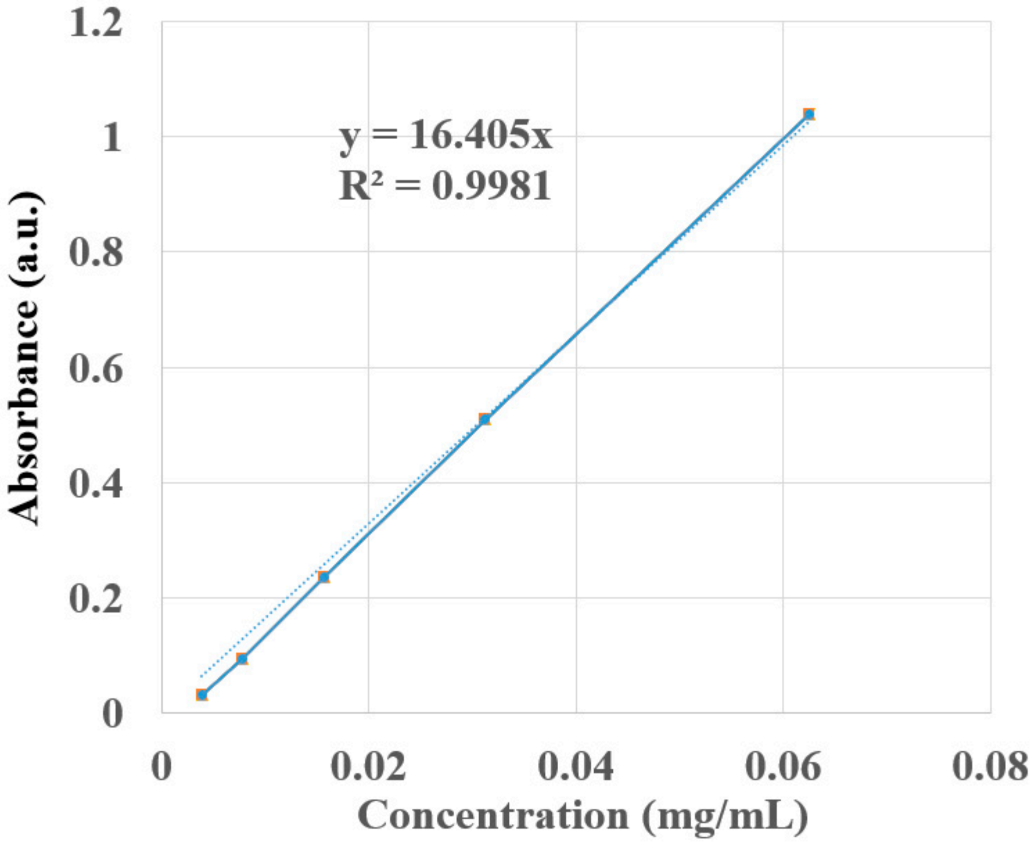

Figure S1. The standard curve for DOX absorbance at 490 nm.

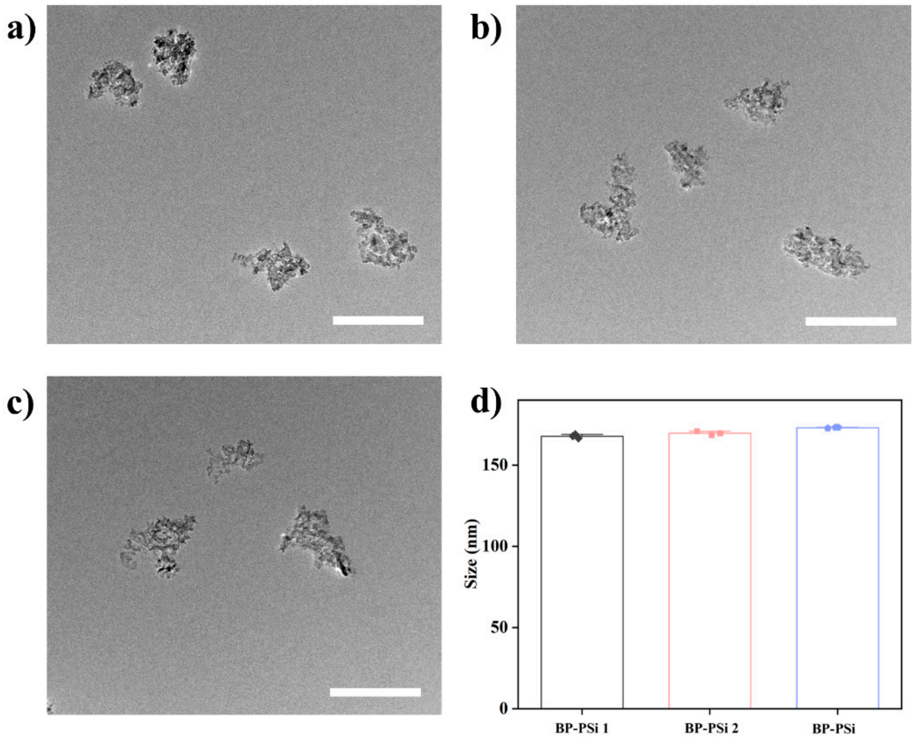

Figure S2. Porous silicon nanoparticles loaded with different amount of bisphosphonates: transmission electron microscopy (TEM) images: (**a**) BP-PSi 1 (mass ratio 0.5), (**b**) BP-PSi 2 (mass ratio 1), (**c**) BP-PSi (mass ratio 2). The scale bar is 200 nm. (**d**) mean diameter. Data represent mean ± SD (n = 3).

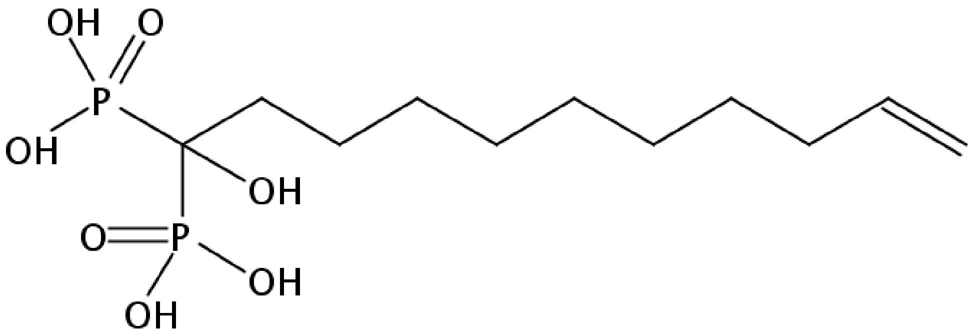

Figure S3. Chemical structure of BP molecule.

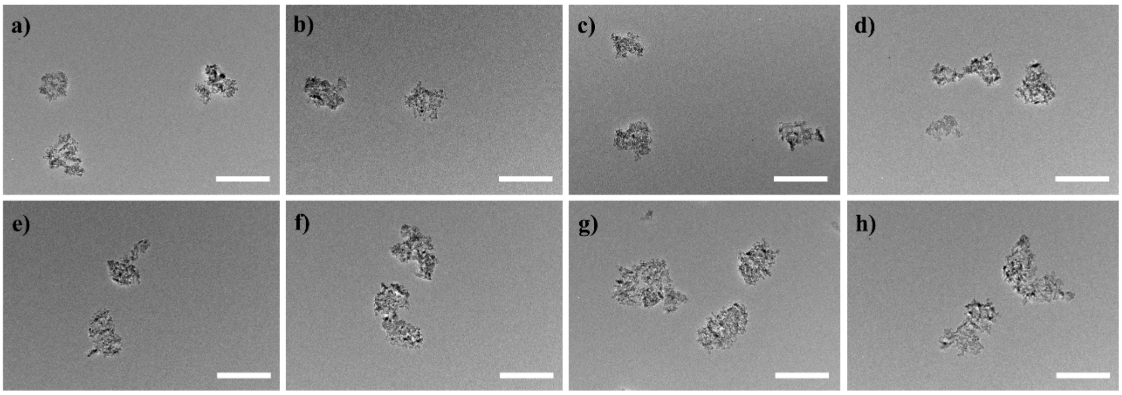

Figure S4. Transmission electron microscopy (TEM) images: (**a**) PSi, (**b**) BP-PSi, (**c**) ^{99m}Tc-BP-PSi, (**d**) ^{68}Ga-BP-PSi, (**e**) PEG-^{99m}Tc-BP-PSi, (**f**) PEG-^{68}Ga-BP-PSi, (**g**) DOX-PEG-^{99m}Tc-BP-PSi, (**h**) DOX-PEG-^{68}Ga-BP-PSi. The scale bar is 200 nm.

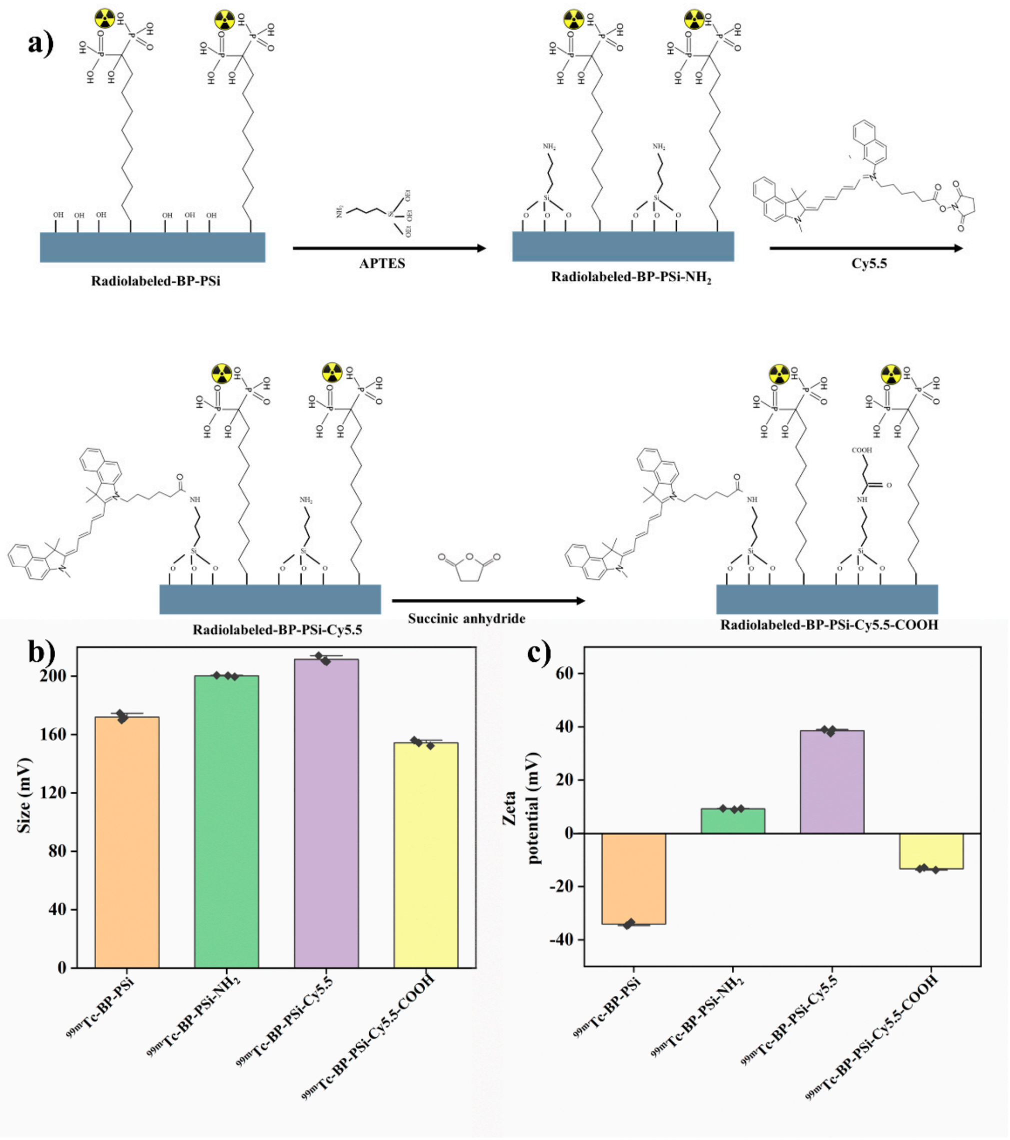

Figure S5. a) Radiolabeled NPs were further modified with 3-aminopropyl triethyl silane (APTES), Cyanine5.5 NHS ester (Cy5.5), and succinic anhydride. (**a**) Scheme, (**b**) Mean diameter, and (**c**) Zeta potential of ^{99m}Tc-BP-PSi, ^{99m}Tc-BP-PSi-NH_2, ^{99m}Tc-BP-PSi-Cy5.5, and ^{99m}Tc-BP-PSi-Cy5.5-COOH NPs.

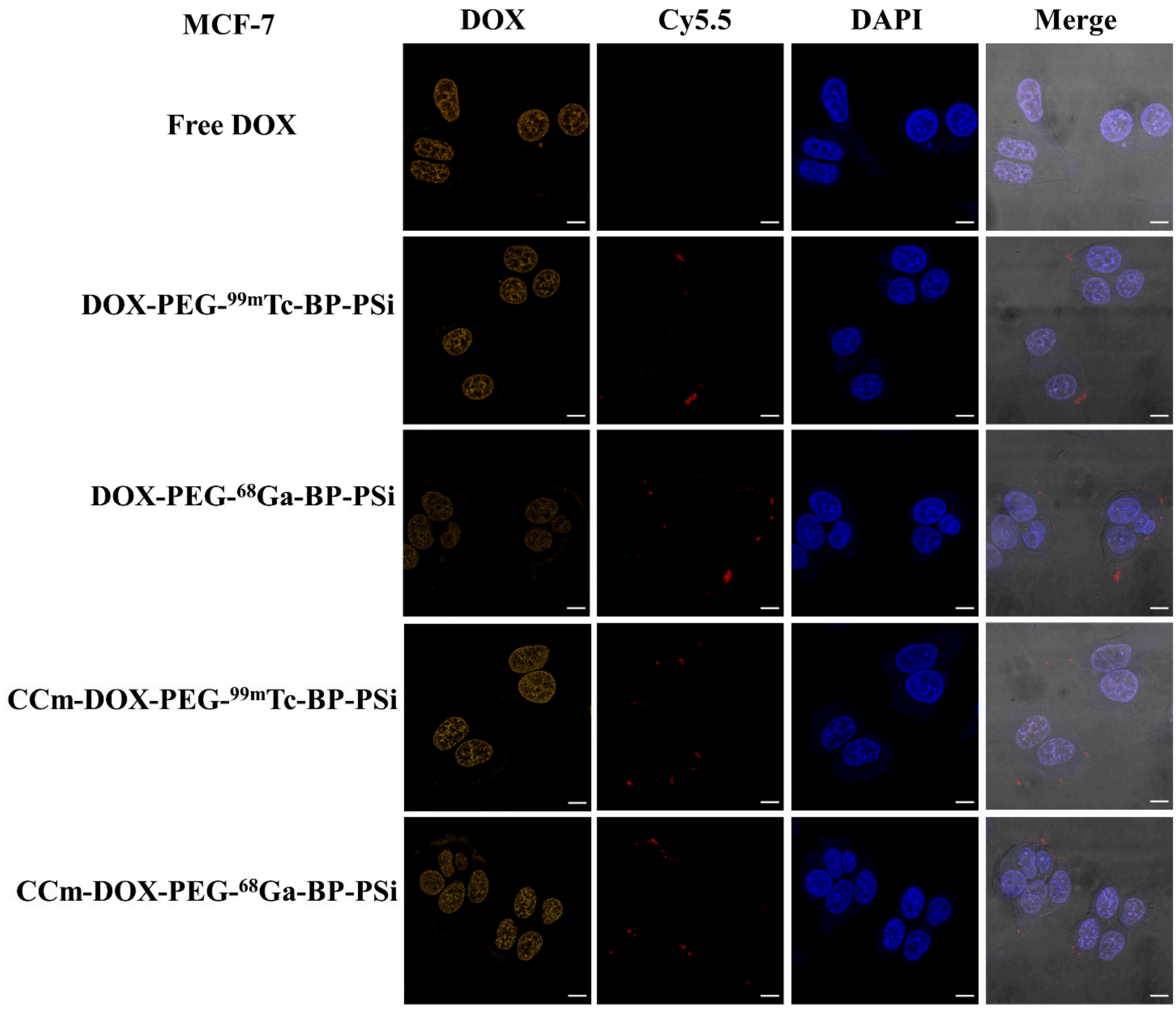

Figure S6. Representative confocal laser scanning microscopy (CLSM) images of MCF-7 cells after 4 h incubation with equivalent 2.5 μg/mL DOX doses in all groups: free DOX, DOX-PEG-^{99m}Tc-BP-PSi, DOX-PEG-^{68}Ga-BP-PSi, CCm-DOX-PEG-^{99m}Tc-BP-PSi, CCm-DOX-PEG-^{68}Ga-BP-PS. The PSi cores and cell nuclei were labeled with Cy5.5 (red) and DAPI (blue), respectively. The scale bar is 10 μm.

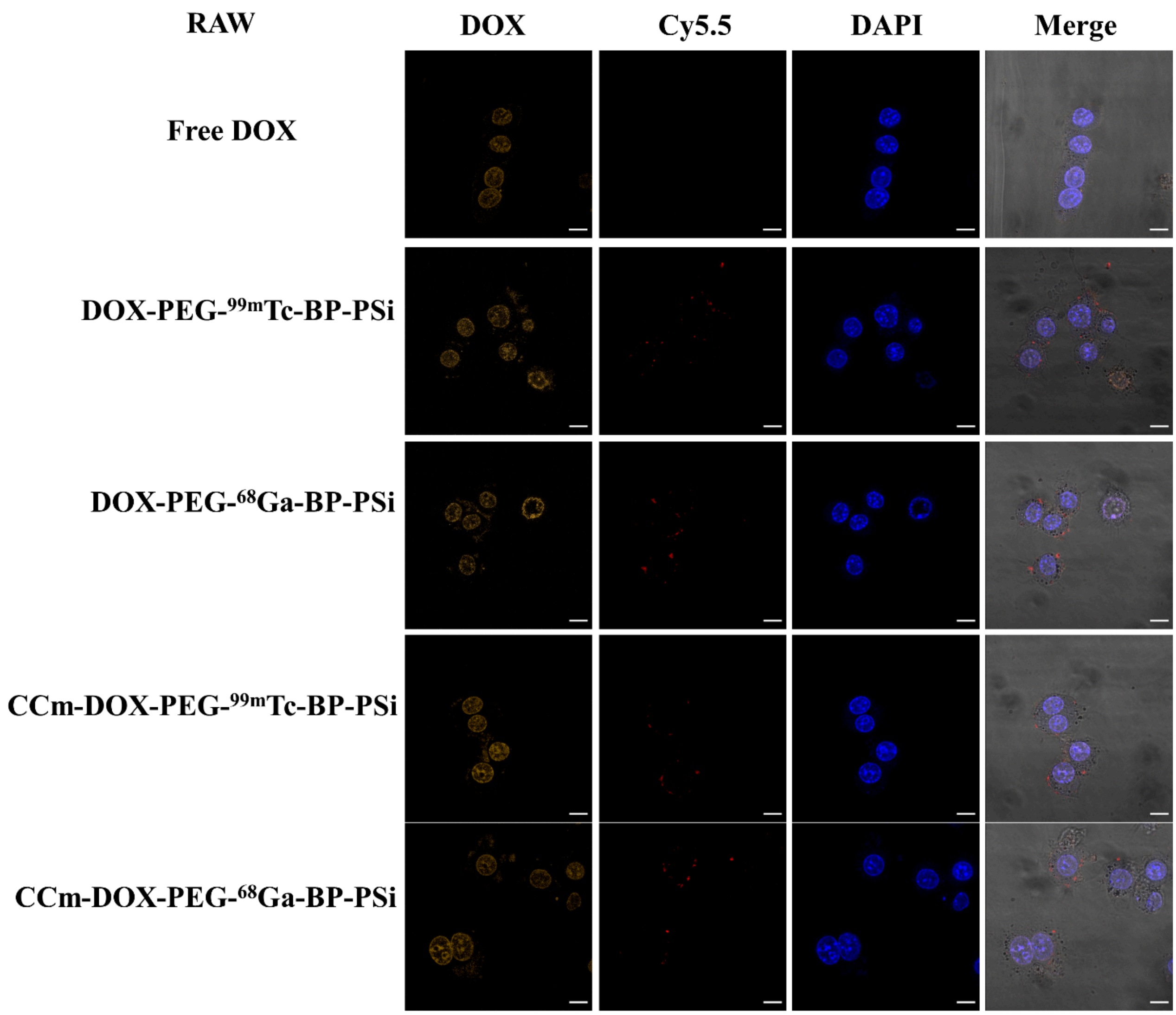

Figure S7. Representative confocal laser scanning microscopy (CLSM) images of RAW267.4 cells after 4 h incubation with equivalent 2.5 μg/mL DOX doses in all groups: free DOX, DOX-PEG-^{99m}Tc-BP-PSi, DOX-PEG-^{68}Ga-BP-PSi, CCm-DOX-PEG-^{99m}Tc-BP-PSi, CCm-DOX-PEG-^{68}Ga-BP-PS. The PSi cores and cell nuclei were labeled with Cy5.5 (red) and DAPI (blue), respectively. The scale bar is 10 μm.

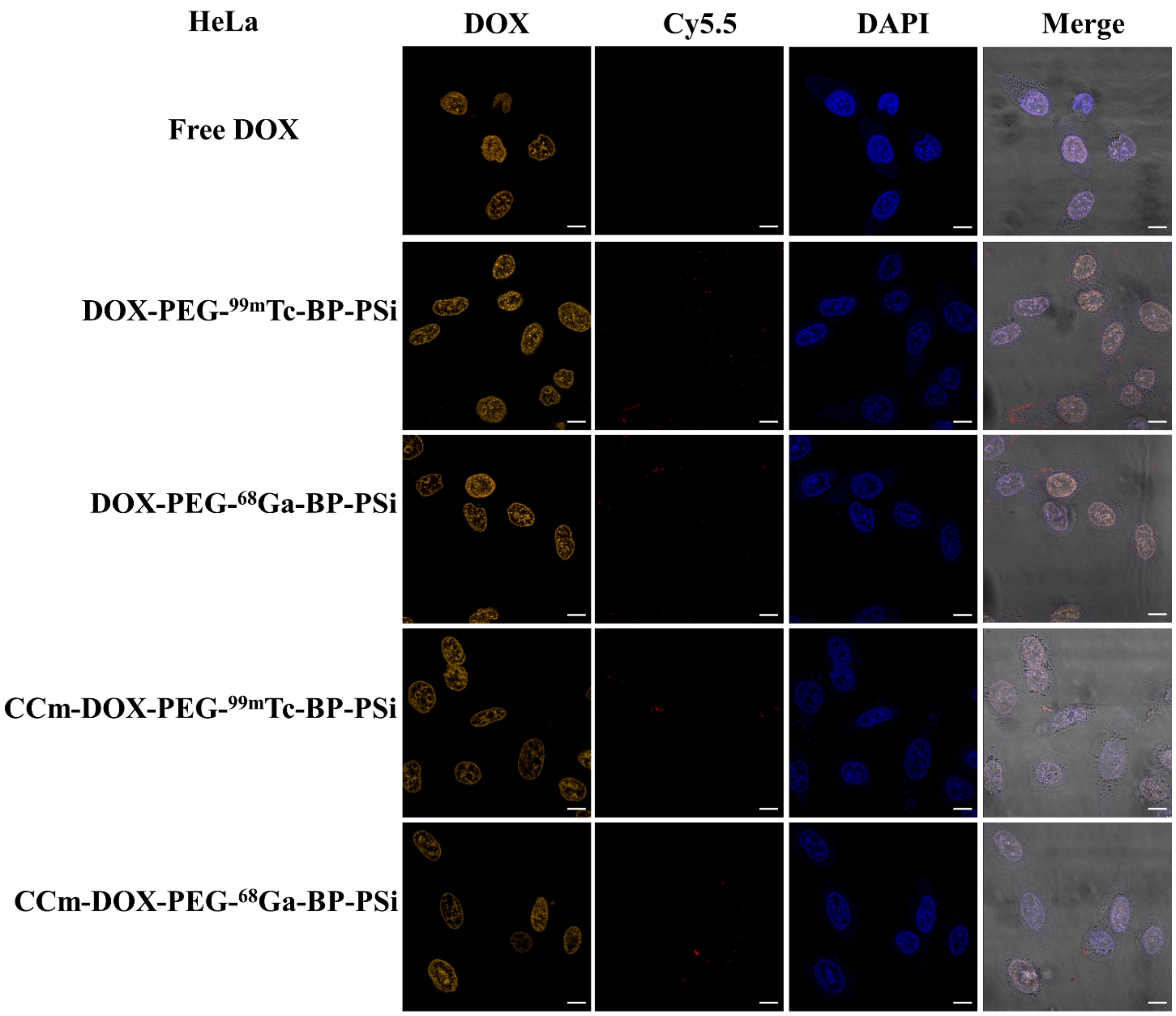

Figure S8. Representative confocal laser scanning microscopy (CLSM) images of HeLa cells after 4 h incubation with equivalent 2.5 μg/mL DOX doses in all groups: free DOX, DOX-PEG-^{99m}Tc-BP-PSi, DOX-PEG-^{68}Ga-BP-PSi, CCm-DOX-PEG-^{99m}Tc-BP-PSi, CCm-DOX-PEG-^{68}Ga-BP-PSi. The PSi cores and cell nuclei were labeled with Cy5.5 (red) and DAPI (blue), respectively. The scale bar is 10 μm.

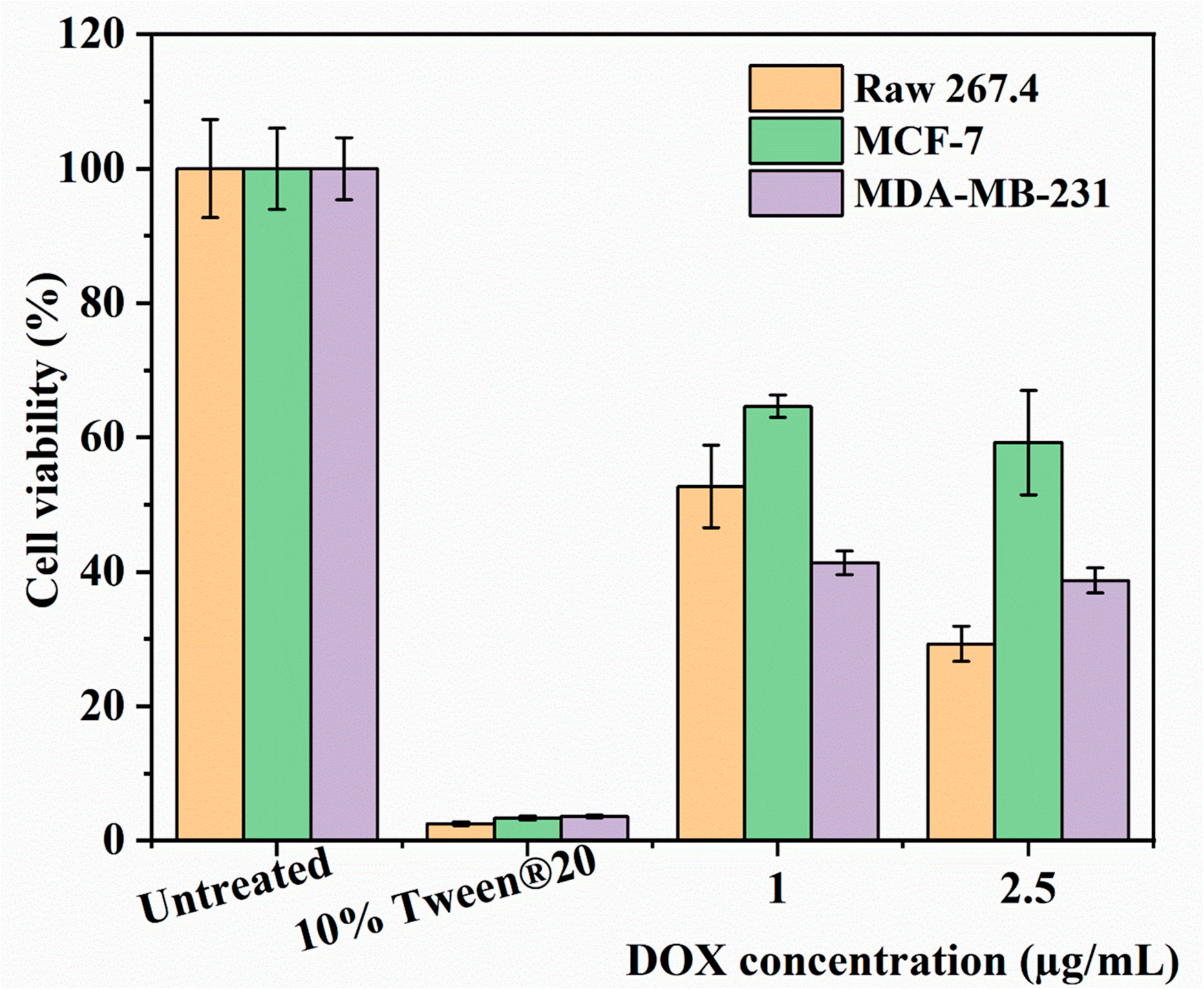

Figure S9. Cell viability of CCm-DOX-PEG-BP-PSi treated Raw 267.4, MCF-7, and MDA-MB-231 cells with equivalent DOX doses at 24 h. Data represent mean ± SD (n = 5). The untreated cells and cells incubated with 10% Tween®20 were used as negative and positive controls, respectively.
